# Synchronous primary carcinoid tumor and primary adenocarcinoma arising within mature cystic teratoma of horseshoe kidney: a unique case report and review of the literature

**DOI:** 10.1186/1746-1596-4-17

**Published:** 2009-06-14

**Authors:** Henry B Armah, Anil V Parwani, Aleksandr M Perepletchikov

**Affiliations:** 1 Department of Pathology, Presbyterian-Shadyside Hospital, University of Pittsburgh Medical Center, Pittsburgh, Pennsylvania, USA; 2 Department of Pathology, Mercy Hospital, University of Pittsburgh Medical Center, Pittsburgh, Pennsylvania, USA

## Abstract

**Background:**

Malignant transformation of mature cystic teratoma is a rare complication. While any of the constituent tissues of a teratoma has the potential to undergo malignant transformation, squamous cell carcinoma is the most commonly associated malignancy. Renal carcinoid tumors are rare and frequently associated with horseshoe kidney and renal teratoma. Renal teratoma rarely presents together with carcinoid tumor or adenocarcinoma. To the best of our knowledge, there has never been a report of renal teratoma coexisting with both carcinoid tumor and adenocarcinoma.

**Methods:**

Here, we present a unique and first case of synchronous primary carcinoid tumor and moderately differentiated adenocarcinoma arising within mature cystic teratoma of horseshoe kidney in a 50-year-old female. Lumbar spine X-ray, done for her complaint of progressive chronic low back pain, accidentally found a large calcification overlying the lower pole of the right kidney. Further radiologic studies revealed horseshoe kidney and a large multiseptated cystic lesion immediately anterior to the right renal pelvis with central calcification and peripheral enhancement. She underwent right partial nephrectomy.

**Results:**

Macroscopically, the encapsulated complex solid and multiloculated cystic tumor with large calcification, focal thickened walls and filled with yellow-tan gelatinous material. Microscopically, the tumor showed coexistent mature cystic teratoma, moderately differentiated adenocarcinoma and carcinoid tumor. Immunohistochemically, alpha-methylacyl-coenzyme A-racemase, calretinin, CD10 and thyroid transcription factor-1 were negative in all the three components of the tumor. The teratomatous cysts lined by ciliated epithelium showed strong staining for cytokeratin 7 and pancytokeratin, and those lined by colonic-like epithelium showed strong staining for CDX2, cytokeratin 20 and pancytokeratin, but both were negative for calretinin. Additionally, the teratomatous cyst wall showed strong staining for smooth muscle actin, and weak staining for carbonic anhydrase IX, CD99, chromogranin and synaptophysin. The adenocarcinoma component was strongly positive for cytokeratin 7 and pancytokeratin, weakly positive for synaptophysin and CD56, and negative for carbonic anhydrase IX, CD99, CDX2, chromogranin, cytokeratin 20 and smooth muscle actin. The carcinoid tumor component was strongly positive for CD56, chromogranin and synaptophysin, weakly positive for pancytokeratin, and negative for carbonic anhydrase IX, CD99, CDX2, cytokeratin 7, cytokeratin 20 and smooth muscle actin. She received no adjuvant therapy and is alive without evidence of disease six months after diagnosis and surgery.

**Conclusion:**

This unique and first case herein presented with synchronous primary carcinoid tumor and primary adenocarcinoma arising within mature cystic teratoma of horseshoe kidney emphasizes the need for thorough sectioning and entire submission for histologic evaluation of mature cystic teratomas, in order to avoid missing multiple additional histogenetically distinct neoplasms.

## Background

Malignant transformation of mature cystic teratoma (MCT) is a rare complication occurring in approximately 1–3% of patients who have mature cystic teratoma [1,2]. Although any of the constituent tissues of a teratoma has the potential to undergo malignant transformation, squamous cell carcinoma is the most commonly associated malignancy [1]. Other reported malignancies arising in MCT include carcinoid tumor, adenocarcinoma, basal cell carcinoma, adenosquamous carcinoma, thyroid carcinoma, sebaceous carcinoma, malignant melanoma, sarcoma and neuroectodermal tumor [2,3]. Primary renal carcinoid tumor is a low grade malignancy with neuroendocrine differentiation, and was first described by Resnick et al in 1966 [4]. Since then less than 100 cases of primary renal carcinoid tumor have appeared in the international medical literature, and are often associated with horseshoe kidney (18–26%), renal teratoma (15%) and polycystic kidney disease (2%) [5-40]. Primary carcinoid tumor arising within mature cystic teratoma of the kidney is rare. Only seven cases of primary carcinoid tumor arising in mature cystic teratoma of the kidney have been reported in the world medical literature to date [9-11,15-17,20], since the association was first described in 1976 by Kojiro et al [9]. The simultaneous occurrence of mature cystic teratoma and adenocarcinoma in the kidney is also rare [41]. To the best of our knowledge, the synchronous presentation in the same kidney of mature cystic teratoma, carcinoid tumor and adenocarcinoma has never been reported in the world medical literature. We present a unique and first case of a 50-year-old female with both primary carcinoid tumor and primary moderately differentiated adenocarcinoma simultaneously arising within mature cystic teratoma of horseshoe kidney. Additionally, we review the world medical literature and discuss the extreme rarity of this combination of primary tumors in the kidney and the probable common histogenesis of these synchronous neoplasms in horseshoe kidney.

## Case presentation

**Figure 1:**
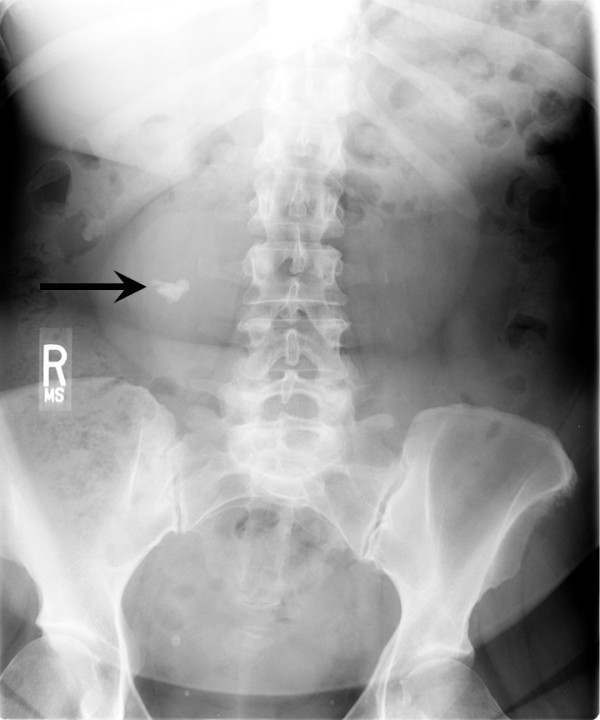
**Anterior-posterior view of lumbar X-ray showing a large calcification (arrow) overlying the lower pole of the right kidney**. R indicates right side.

**Figure 2:**
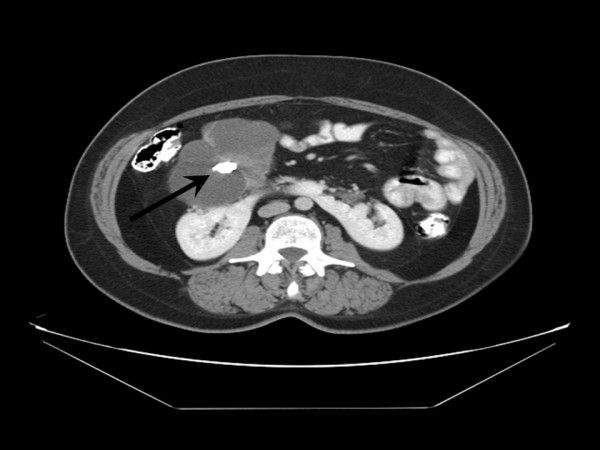
**Computed tomography scan of abdomen and pelvis demonstrating horseshoe shaped kidney and a large multiseptated cystic lesion immediately anterior to the right renal pelvis with central calcification (arrow)**.

The patient was a 50-year-old female who presented with a 3-months history of progressive chronic low back and right hip pain. She had no symptoms of carcinoid syndrome. She had no previous history of malignancy, chemotherapy or radiotherapy. General physical exam was unremarkable. Chest radiographs and electrocardiogram were within normal limits. Her routine hemogram, urine and blood biochemical analyses were within normal ranges. A lumbar spine X-ray, done to workup her complaint of low back pain, accidentally found a large (1.9 cm) calcification overlying the lower pole of the right kidney (Figure 1). Subsequent computed tomography (CT) and magnetic resonance imaging (MRI) scans of abdomen and pelvis revealed horseshoe shaped kidney and a large (10.5 × 7.8 cm) multiseptated cystic lesion immediately anterior to the right renal pelvis with central calcification (1.9 cm) and peripheral enhancement (Figure 2), which was interpreted radiologically as a Bosniak category IV renal lesion with a 90% chance of malignancy. No additional lesions were identified in the brain, gastrointestinal tract, liver, spleen, pancreas, bladder, uterus, ovaries and bones on additional MRI scans of the abdomen, brain, chest and pelvis, and positron emission tomography (PET) scan. This excluded the possibility of other primary malignancies metastatic to the right kidney. Her serum levels of alpha-fetoprotein (α-FP), beta-subunit of human chorionic gonadotropin (β-hCG), carbohydrate antigen (CA) 19-9, CA72-4, CA125, carcinoembryonic antigen (CEA), chromogranin and serotonin were within normal ranges. Her urinary 5-hydroxyindoleacetic acid (5-HIAA) level was also within normal range. In view of the high concern for primary malignancy in this Bosniak category IV right renal lesion, the patient elected to undergo right partial nephrectomy for definitive surgical treatment. After the peritoneal cavity was surgically opened, a thorough inspection and palpation of the peritoneal cavity was performed which revealed no ascitic fluid, adhesions, or other evidence of metastatic disease or non-renal primary malignancy. The entire right renal tumor was surgically resected with an excellent margin of 0.5 cm of normal parenchyma surrounding the entire cyst wall, and the tumor was entirely confined to the kidney. No other lesions were present in the adjacent renal parenchyma. The postoperative period was uneventful and the patient was discharged four days after surgery.

## Methods

### Histologic examination

Tissues were fixed in 10% buffered formalin solution and embedded in paraffin blocks. Four-micrometer-thick sections were obtained and stained with hematoxylin and eosin for microscopic examination.

### Immunohistochemical analysis

Additional sections were used to perform immunohistochemical studies using an avidin-biotin peroxidase technique with hematoxylin counterstain. The antibodies used in this study included the following; alpha-methylacyl-coenzyme A-racemase (AMACR/P504S) [1:100, Ventana Medical Systems Inc, Tucson, Arizona], calretinin (1:100, Becton Dickinson, San Jose, California, USA), carbonic anhydrase IX (CA-IX, 1:200, DakoCytomation, Carpinteria, California, USA), CD10 (1:200, Ventana), CD56 (1:200, DakoCytomation), CD99 (1:100, Ventana), CDX2 (1:100, Ventana), chromogranin (1:200, Signet Pathology Systems Inc, Dedham, Massachusetts, USA), cytokeratin 7 (CK7, 1:200, Ventana), cytokeratin 20 (CK20, 1:200, DakoCytomation), pancytokeratin cocktail (AE1-AE3, 1:500, DakoCytomation; CAM 5.2, 1:50, Becton Dickinson; MNF116, 1:50, DakoCytomation; and UCD/PR-10.11, 1:25, Zymed, San Francisco, California), smooth muscle actin (SMA, 1:200, DakoCytomation), synaptophysin (1:100, DakoCytomation), and thyroid transcription factor-1 (TTF-1, 1:250, Ventana). Appropriate tissue sections that had been shown to be positive or negative for each marker were used as controls. Slides stained omitting the primary antibody were also used as negative controls.

We analyzed the intensity and immunoreactivity of the immunostained sections. The intensity was graded qualitatively as weak, moderate, or strong on the basis of the brown color produced by the 3, 3'-diaminobenzidine chromogen. Diffuse and intense brown staining of the cytoplasmic or nuclear surfaces, as appropriate for each stain, was interpreted as strong staining intensity. Moderate intensity staining was characterized as non-diffuse but intense staining pattern, whereas weak intensity had a non-diffuse and non-intense staining pattern. Immunoreactivity was quantitatively estimated by the percentage of positive cells per representative section. Immunoreactivity was graded as 1+, 2+, and 3+, corresponding to less than 25%, between 25% and 50%, and greater than 50% of neoplastic cells showing positive staining per representative section, respectively.

## Results

### Macroscopic findings

Grossly, the partial nephrectomy specimen revealed an encapsulated multiloculated gray-tan tumor (9.7 × 7.4 × 7.8 cm). The interface between tumor and uninvolved kidney was sharp. Sectioning of the tumor revealed circumscribed complex solid and multiloculated cystic lesion with large area of calcification (1.7 cm). The largest cyst measured up to 1.4 cm, and the cysts were filled with yellow-tan gelatinous material. The wall of the cysts showed focal thickened firm and hard calcified areas. The tumor was confined to the kidney and was at least 0.5 cm from the nearest stapled resection margin. There was no gross evidence of extension of the tumor into the renal pelvis, renal vein, renal artery, ureter, renal sinus, renal capsule, or perinephric adipose tissue. The entire tumor was submitted for histological examination.

### Microscopic findings

**Figure 3:**
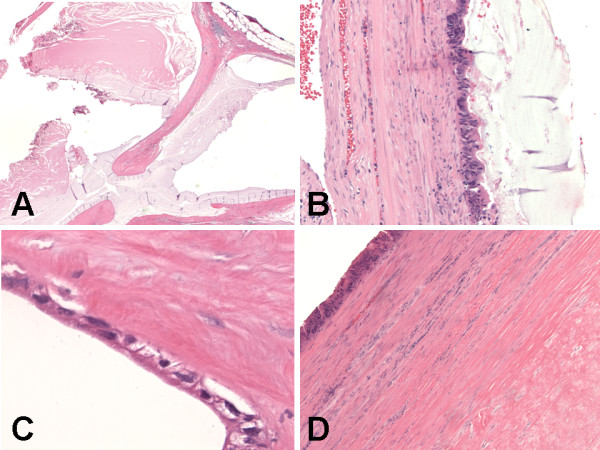
**Histologic (hematoxylin and eosin stain) findings of synchronous primary carcinoid tumor and primary adenocarcinoma arising within mature cystic teratoma of horseshoe kidney**. (A) Teratomatous component with multilocular cystic spaces. Original magnification ×20. (B) Teratomatous cyst lined by mucinous columnar enteric-type or colonic-like epithelium with muscular wall. Original magnification ×200. (C) Teratomatous cyst lined by ciliated epithelium with muscular wall. Original magnification ×600. (D) Teratomatous cyst wall composed predominantly of smooth muscle. Original magnification ×100.

**Figure 4:**
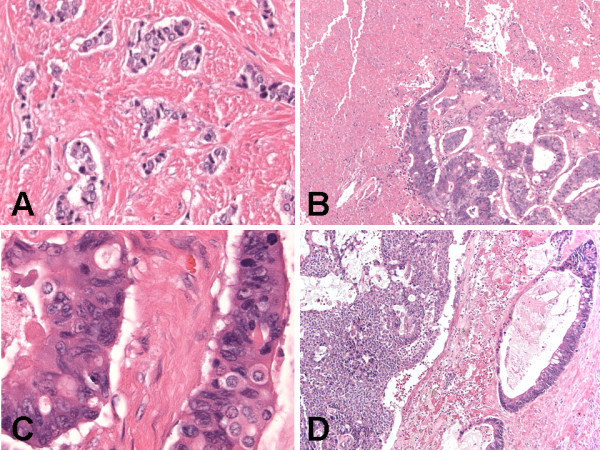
**Histologic (hematoxylin and eosin stain) findings of synchronous primary carcinoid tumor and primary adenocarcinoma arising within mature cystic teratoma of horseshoe kidney**. (A) Adenocarcinoma component with infiltrating atypical glands. Original magnification ×200. (B) Adenocarcinoma component composed of cohesive pleomorphic epithelioid cells with adjacent necrosis. Original magnification ×100. (C) Adenocarcinoma component showing large cells with abundant eosinophilic cytoplasm, enlarged nuclei with contour irregularities, and occasional prominent nucleoli. Original magnification ×600. (D) Carcinoid tumor (left part of figure) and adenocarcinoma (right part of figure) components adjacent and closely apposed to each other with a clear transition zone. Original magnification ×100.

**Figure 5:**
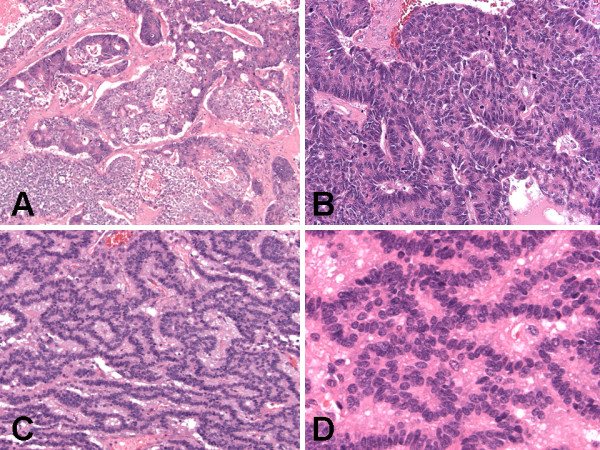
**Histologic (hematoxylin and eosin stain) findings of synchronous primary carcinoid tumor and primary adenocarcinoma arising within mature cystic teratoma of horseshoe kidney**. (A) Carcinoid tumor (left lower part of figure) and adenocarcinoma (right upper part of figure) components adjacent and closely apposed to each other without a clear transition zone. Original magnification ×100. (B) Carcinoid tumor showing the classical architectural pattern of trabecular nests of monotonous small round cells with peripheral palisading. Original magnification ×200. (C) Carcinoid tumor showing the classical architectural pattern of anastomosing ribbon-like nests of monotonous small round cells. Original magnification ×200. (D) Carcinoid tumor showing the classical cytologic features of fine granular "salt-and-pepper" chromatin pattern. Original magnification ×600.

The histological examination revealed three components. The first component was multilocular cystic spaces (Figure 3A) lined by mucinous columnar enteric-type or colonic-like epithelium (Figure 3B) and ciliated epithelium (Figure 3C), and containing fragments of smooth muscle (Figure 3D) in the wall. These findings in the first component represent a mature cystic teratoma (Figures 3A–D). The predominant teratomatous element in the first component was mucinous columnar enteric-type or colonic-like epithelium (Figure 3B), followed by ciliated epithelium (Figure 3C), and smooth muscle (Figure 3D). The second and third components of the tumor make up the solid parts of the tumor. The second component of the tumor showed tightly-packed back-to-back highly atypical infiltrating glands (Figure 4A) composed of cohesive pleomorphic epithelioid cells with adjacent necrosis (Figure 4B). The tumor cells were large with abundant eosinophilic cytoplasm, enlarged nuclei with contour irregularities, and occasional prominent nucleoli (Figure 4C). Mitotic figures were readily identified. There was no evidence of lymphovascular invasion. These features of the second component represent an invasive moderately differentiated adenocarcinoma (Figures 4A–D &5A). The carcinoid tumor and adenocarcinoma components were found adjacent and closely apposed to each other, with (Figures 4D) or without (Figures 5A) a clear transition zone. The third component showed proliferating trabecular (Figure 5B) and anastomosing ribbon-like (Figure 5C) nests of monotonous small round cells with fine granular "salt-and-pepper" chromatin pattern (Figure 5D), and peripheral palisading (Figure 5B). Mitotic figures were not identified. There was no evidence of lymphovascular invasion. These features of the third component represent a carcinoid tumor (Figures 4D &5A–D). The carcinoid tumor and adenocarcinoma components were found underneath and closely apposed to the epithelial lining of the teratomatous cysts, and focally invading teratomatous smooth muscle wall (Figures 4A). All surgical resection margins were free of the three components of the tumor.

### Immunohistochemical findings

**Figure 6:**
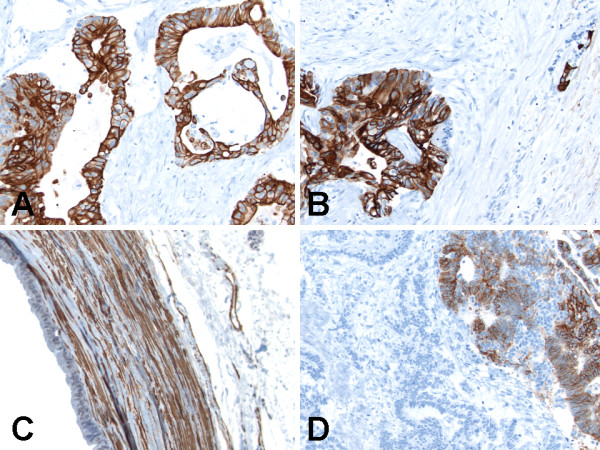
**Immunohistochemical (immunoperoxidase and hematoxylin counterstain) findings of synchronous primary carcinoid tumor and primary adenocarcinoma arising within mature cystic teratoma of horseshoe kidney**. (A) CDX2 staining was positive in the mucinous columnar enteric-type or colonic-like epithelium lining of teratomatous cysts. Original magnification ×200. (B) Cytokeratin 20 staining was positive in the mucinous columnar enteric-type or colonic-like epithelium lining of teratomatous cysts. Original magnification ×200. (C) Smooth muscle actin was positive in teratomatous cyst wall. Original magnification ×200. (D) Cytokeratin 7 staining was positive in the adenocarcinoma component in right upper part of figure, but negative in the carcinoid tumor component in left lower part of figure. Original magnification ×200.

**Figure 7:**
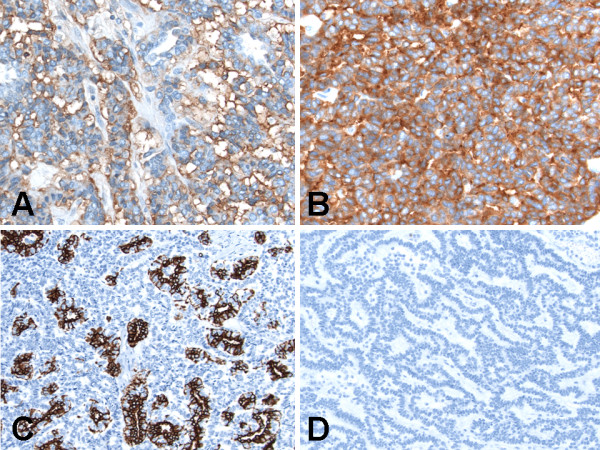
**Immunohistochemical (immunoperoxidase and hematoxylin counterstain) findings of synchronous primary carcinoid tumor and primary adenocarcinoma arising within mature cystic teratoma of horseshoe kidney**. (A) CD56 staining was positive in the carcinoid tumor component. Original magnification ×400. (B) Synaptophysin staining was positive in the carcinoid tumor component. Original magnification ×400. (C) Pancytokeratin staining was focally positive in the carcinoid tumor component. Original magnification ×200. (D) Cytokeratin 20 was negative in the carcinoid tumor component. Original magnification ×200.

Alpha-methylacyl-coenzyme A-racemase (AMACR/P504S), calretinin, CD10 and thyroid transcription factor-1 (TTF-1) did not stain any of the three components of the tumor. The teratomatous cysts were lined by ciliated epithelium (strong and diffuse cytoplasmic labeling for cytokeratin 7 and pancytokeratin) and mucinous columnar enteric-type or colonic-like epithelium (strong and diffuse cytoplasmic labeling for CDX2 [Figure 6A], cytokeratin 20 [CK20, Figure 6B] and pancytokeratin), but were negative for calretinin (a mesothelial marker). Additionally, the teratomatous cyst wall was strongly and diffusely immunoreactive (3+, cytoplasmic staining) for smooth muscle actin (SMA, Figure 6C); and weakly and focally immunoreactive (1+, cytoplasmic staining) for carbonic anhydrase IX (CA-IX), CD99, chromogranin and synaptophysin. The carcinoid tumor and adenocarcinoma components were found adjacent and closely apposed to each other (Figure 6D), with the adenocarcinoma component in right upper part of figure being strongly and diffusely immunoreactive (3+, cytoplasmic staining) for CK7 whilst the carcinoid tumor component in left lower part of figure being negative for CK7 (Figures 6D). The adenocarcinoma component was strongly and diffusely immunoreactive (3+, cytoplasmic staining) for CK7 (Figure 6D) and pancytokeratin; weakly and focally immunoreactive (1+, cytoplasmic staining) for CD56 and synaptophysin; and negative for CA-IX, CD99, CDX2, chromogranin, CK20 and SMA. The carcinoid tumor component was strongly and diffusely immunoreactive (3+, cytoplasmic staining) for CD56 (Figure 7A), chromogranin and synaptophysin (Figure 7B); weakly and focally immunoreactive (1+, cytoplasmic staining) for pancytokeratin (Figure 7C); and negative for CA-IX, CD99, CDX2, CK7, CK20 (Figure 7D) and SMA.

### Treatment, follow-up and prognosis

On the basis of the above histopathological and immunohistochemical features, a definitive diagnosis of synchronous primary carcinoid tumor and primary moderately differentiated adenocarcinoma arising within mature cystic teratoma of horseshoe kidney was rendered. This tumor could not be staged since no relevant staging system exists for synchronous primary carcinoid tumor and primary moderately differentiated adenocarcinoma of kidney. Her postsurgery serum levels of alpha-fetoprotein (α-FP), beta-subunit of human chorionic gonadotropin (β-hCG), carbohydrate antigen (CA) 19-9, CA 72-4, CA 125, carcinoembryonic antigen (CEA), chromogranin and serotonin continued to remain within normal ranges. Her postsurgery urinary 5-HIAA level also continued to remain within normal range. Additionally, postsurgery whole body somatostatin receptor scintigraphy with octreotide performed was negative, hence confirming the carcinoid tumor as originating primarily in the resected renal tumor (rather than elsewhere with metastasis to the kidney) and the absence of metastases from the resected renal carcinoid tumor. Hence, the partial nephrectomy with complete resection of the tumor was considered adequate conservative clinical management. She received no adjuvant therapy. Two follow-up CT and MRI scans of the brain, abdomen, chest and pelvis, and PET scans performed at 3-month intervals after surgery revealed no lesions. The patient herein presented is alive with no evidence of local recurrence or metastatic disease, six months postoperatively, and regular periodic follow-up with interval CT, MRI and PET imaging studies, and octreotide scintigraphy is planned.

## Discussion

Mature cystic teratomas may consist of a wide variety of ectodermal, mesodermal, and endodermal tissues, and hence the possibility of additional neoplasms occurring in teratomas, though rare, can at least be anticipated. It is not surprising that squamous cell carcinoma leads the list of secondary neoplasms arising in teratomas, because many mature cystic teratomas contain large amounts of squamous epithelium [1-3]. In second place among secondary neoplasms in mature cystic teratomas, carcinoid tumor and adenocarcinoma are said to be of equal frequency, but the former is reported to be increasing in frequency [1-3]. Hence, adenocarcinoma or carcinoid tumor arising from mature cystic teratoma of kidney is an uncommon occurrence [9-11,15-17,20,41]. The simultaneous occurrence of mature cystic teratoma and adenocarcinoma in the same kidney is rare [41], and so is the simultaneous occurrence of mature cystic teratoma and carcinoid tumor in the same kidney [9-11,15-17,20]. Similarly, the simultaneous occurrence of carcinoid tumor and adenocarcinoma in the same kidney is rare. To the best of our knowledge, the synchronous presentation in the same kidney of these three neoplasms (mature cystic teratoma, carcinoid tumor and adenocarcinoma) has never been reported in the world medical literature. We have presented a unique and first case of a 50-year-old female with both carcinoid tumor and adenocarcinoma simultaneously arising within mature cystic teratoma of horseshoe kidney. We discuss below the extreme rarity of this combination of three primary tumors in the kidney and the probable common histogenesis of these three synchronous neoplasms in horseshoe kidney.

Carcinoid tumors are characteristically low grade malignant tumors with neuroendocrine differentiation that have been described in several locations, including the gastrointestinal, respiratory, hepatobiliary, and genitourinary systems. Carcinoid tumors most commonly occur in the gastrointestinal tract (74%) and bronchial system of the lungs (25%) [5,7,18,19,21,24,33,42,43]. In less than 1% of cases these tumors have been reported in the genitourinary system. Carcinoid tumors arising in the genitourinary system are rare, but have been reported in the ovary, testes, kidney and prostate [3,5,7,18,19,21,24,33,42,43]. Primary renal carcinoid tumor is the second most prevalent genitourinary carcinoid tumor in each sex, following testicular carcinoids in males and ovarian carcinoids in females [5,7,18,19,21,24,33,42,43]. Primary renal carcinoid tumors are among the most unusual of all renal neoplasms, since neuroendocrine cells are not found within normal renal parenchyma. Primary renal carcinoid tumors have been reported in the literature primarily as case reports (as in the case herein presented), with the three largest series to date consisting of 5 [5], 6 [21], and 21 [7] patients. Primary carcinoid tumor of the kidney was first described by Resnick et al. in 1966 [4], and since then fewer than 100 cases of primary renal carcinoid tumor have appeared in the international medical literature [5-40]. Primary renal carcinoid tumors have been reported to arise most commonly in the setting of acquired and congenital renal abnormalities (as in the case herein presented), such as horseshoe kidney (18–26%) [7,8,12-14,18,20,21,38], renal teratoma or teratoid malformation (15%) [9-11,15-17,20], and polycystic kidney disease (2%) [40]. The age range for reported cases of primary renal carcinoid tumor is 12 to 78 years, but most patients present in the fourth to seventh decades of life and there is no gender predilection [5-40]. A recent review of 56 primary renal carcinoid tumors reported in the literature up to December 2005 by Romero et al. [18] observed that the median patient age was 49 years. Romero et al. [18] further reported that horseshoe kidneys were present in 17.8% of patients (as in the case herein presented), incidental diagnosis was made in 28.6% of patients (as in the case herein presented), and metastases were present in 45.6% of patients at initial diagnosis. They also observed that the significant adverse prognostic factors include age greater than 40 years, tumor size greater than 4 cm, purely solid tumors on cut surface, mitotic rate higher than 1 per 10 high power fields, metastasis at initial diagnosis and tumors extending through the renal capsule [18]. The largest series of primary renal carcinoid tumors reported to date consisting of 21 patients by Hansel et al. [7] observed that the mean patient age was 52 years. As in the case herein presented, Hansel et al. [7] further reported that horseshoe kidneys were present in 19% of patients, and calcifications were present in 23.8% of cases of primary renal carcinoid tumor. They concluded that primary renal carcinoid tumor was morphologically and immunohistochemically similar to carcinoid tumors present at other anatomic sites, and that patients frequently presented with regional lymph node metastases and may progress to distant organ metastases, but usually have a prolonged clinical course despite widely metastatic disease [7]. Complete surgical resection is the main treatment modality for primary renal carcinoid tumors and can be curative for localized disease (as in the case herein presented), and long-term follow-up of patients is recommended since metastases have occurred as late as seven years after the primary diagnosis [7,18,21].

Primary carcinoid tumors of the kidney are rare [5-40], and primary carcinoid tumor arising within horseshoe kidneys [7,8,12-14,18,20,21,38] and mature teratomas of the kidney [9-11,15-17,20] are even rarer. Less than 100 cases of carcinoid tumor of the kidney have been reported in the international medical literature [5-40], including 18 cases arising in horseshoe kidneys [7,8,12-14,18,20,21,38] and seven cases arising within mature cystic teratomas [9-11,15-17,20]. A recent review by Armah and Parwani [20] identified that only seven cases of primary carcinoid tumor arising in mature cystic teratoma of kidney have been reported in the world medical literature to date [9-11,15-17,20], since the association was first described in 1976 by Kojiro et al [9]. Primary carcinoid tumor arising in a mature teratoma of the kidney is over-represented in patients with congenital developmental renal defects such as horseshoe kidney, with one out of the seven cases (15%) of primary carcinoid tumor arising in mature cystic teratoma occurring in a horseshoe kidney [9-11,15-17,20]. Epidemiologically, primary carcinoid tumor arising within mature cystic teratoma of the kidney occurred predominantly in the fourth to seventh decades of life (mean age of 41.4 years), except one case occurring at age 23, and showed no sex predilection [20]. An incidental diagnosis was made in 28.6% of cases, and clinically apparent carcinoid syndrome was absent in all the seven reviewed cases of primary carcinoid tumor arising within mature cystic teratoma of the kidney [20] (as in the case herein presented), likely reflecting their hindgut origin [13,22,39] and the breakdown of their secreted biologically active hormones in the liver before reaching the systemic arterial circulation. Armah and Parwani further observed that primary carcinoid tumor arising within mature cystic teratoma of the kidney were morphologically similar to carcinoid tumors present in normal kidneys and at other anatomic sites, and that surgery was curative with no additional treatment required, and no local recurrences and metastases occurred in all seven cases reviewed [20], as in the case herein presented. Immunohistochemically, synaptophysin, chromogranin and neuron specific enolase were the most valuable markers for the diagnosis of primary carcinoid tumor arising within mature teratoma of the kidney [7,18,20,21], as in the case herein presented. Despite the absence of long term follow-up data for some of the seven cases reviewed, the biologic behavior and prognosis of primary carcinoid tumor arising within mature teratoma of the kidney appeared excellent [20], as in the case herein presented. Recent studies comparing the prognosis of carcinoid tumors arising within teratomatous and normal kidneys have reported contradictory findings [8,12,18,20,33]. Four studies have reported better prognosis for carcinoid tumor arising within teratomatous kidneys compared to those arising within normal kidneys [8,12,20,33]. However, a recent review of renal carcinoid tumors revealed that neither renal teratoma nor horseshoe kidneys derived carcinoid tumors were associated with a better prognosis than carcinoid tumors originating in normal kidneys [18]. Hence, although definitive conclusions cannot be drawn from such a small set of studies and patients without systematic long term 5-year follow-up, the ultimate biologic behavior of primary carcinoid tumor arising within mature teratoma of the kidney and horseshoe kidneys may be excellent, and may be better than that for carcinoid tumors arising in normal kidneys and non-renal locations [8,12,20,33].

We were not able to find any reported case of simultaneous occurrence of mature cystic teratoma, carcinoid tumor and adenocarcinoma in the same kidney, in our literature search. We have herein described a case of coexistent mature cystic teratoma, carcinoid tumor and adenocarcinoma in a horseshoe kidney in a 50-year-old female. The age of the case herein presented is close to the mean and/or median age of both previously reported patients with carcinoid tumor arising within normal kidneys (49–52 years) [7,18] and carcinoid tumor arising within teratomatous kidneys (41.4 years) [20]. The strong expression of three neuroendocrine markers (CD56, chromogranin and synaptophysin) and the absence of expression of two epithelial markers (CK7 and CK20), exclude the possibility of invasive urothelial carcinoma arising from the renal pelvis in the case herein presented. Most urothelial carcinomas have the immunoprofile of CK7+/CK20+ or CK7+/CK20- [44]. In contrast, most carcinoid tumors arising from the digestive tract or of hindgut origin have the pattern CK7-/CK20- [44,45], as in the case herein presented. Therefore, it is most likely that the carcinoid tumor in the present case arose outside the urothelial epithelium of the renal pelvis.

Carcinoid tumors are thought to arise from enterochromaffin cells or amine precursor uptake and decarboxylation (APUD) cells, and are widely distributed throughout the body. In the urogenital tract, APUD cells have been described in the urinary bladder (especially in the neck and trigone), the urethra, the prostate, and the renal pelvis, but not in the renal parenchyma [19,33,42,43,46,47]. In contrast, paraganglionic tissue is present in fetal or adult renal hilum [47-49]. Although primary renal carcinoid tumors, as in the case herein presented, exhibit morphologic and immunohistochemical features consistent with a hindgut neuroendocrine phenotype [13,22,39], the precise histogenesis of renal carcinoid tumors is uncertain and is a matter for continuing speculation. Multiple published reports support the notion that renal carcinoid tumors are derived from interspersed neuroendocrine cells associated with acquired and congenital renal abnormalities such as teratomas [9-11,15-17,20] and horseshoe kidneys [7,8,12-14,18,20,21,38]. Carcinoid tumors occurring in renal teratomas are thought to be derived from neuroendocrine cells of the gastrointestinal and respiratory epithelium, which are components of these teratomatous lesions [15]. Two main hypotheses have been proposed for the coexistence of congenital and acquired renal abnormalities (such as horseshoe kidney and mature cystic teratoma) and secondary malignancies (such as carcinoid tumor and adenocarcinoma in the case herein presented) in the kidney. The most popular hypothesis, the totipotent cell hypothesis, states that primary renal carcinoid tumor arise from totipotential primitive stem cells capable of neuroendocrine, mesenchymal and epithelial differentiation [5,7,10,15,18,20,21,24,34,37]. Although conclusive evidence for this theory is lacking at present, one renal carcinoid tumor has been shown to share some genetic aberrations with renal cell carcinomas, indicating a common genetic event in the tumorigenesis for these two entities [34]. Furthermore, this hypothesis of derivation from a multipotent stem cell is most consistent with the occurrence of neuroendocrine tumor in conjunction with epithelial malignancies, which might express markers of both components, as in the case herein presented.

The less popular hypothesis states that the coexistence of carcinoid tumors (and other secondary malignancies such as adenocarcinoma in the case herein presented) with congenital and acquired renal abnormalities (such as horseshoe kidney and mature cystic teratoma) are due to hyperplasia of interspersed neuroendocrine cells within metaplastic or teratomatous epithelium in horseshoe kidneys; or nests of misplaced progenitor cells developing into teratomatous intestinal or respiratory epithelia in renal teratomas, might serve as a nidus for renal carcinoid tumors and adenocarcinoma. Cases of renal carcinoid tumor arising in association with renal teratoma and/or horseshoe kidneys lend weight to this hypothesis [5,7,10,15,18,20,21,24,34,37]. The relative risk of renal carcinoid tumors in patients with horseshoe kidneys has been calculated at between 62 and 85 [12,13]. Horseshoe kidneys have been proposed to be the result of teratogenic factors, which may also account for the increased risk of malignant tumors in horseshoe kidneys [11-13]. The high association of carcinoid tumors with horseshoe kidneys is likely due to predisposing embryological factors or teratogenic events involving the abnormal migration of posterior nephrogenic cells *in utero*, which coalesce to form the isthmus of horseshoe kidneys [12]. Additionally, this hypothesis is supported by the common occurrence of renal carcinoid tumors in the isthmus of teratomatous [9-12,15-17,20] and horseshoe kidneys [7,8,12-14,18,20,21,38], as in the case herein presented with an additional adenocarcinoma. Applying this hypothesis to the case herein presented, the carcinoid tumor and adenocarcinoma would represent secondary tumors derived from foci of neuroendocrine and epithelial differentiation in the mature cystic teratoma of the horseshoe kidney. This hypothesis suggests that renal carcinoid tumors bearing no relationship to congenital and acquired renal abnormalities might arise directly from neuroendocrine cells situated in the renal pelvic urothelium [19,33,42,43,46,47]. From the above discussion, the direct supporting experimental and clinical evidence for these two hypotheses of histogenesis for multiple malignancies arising within teratomatous and horseshoe kidneys is inconclusive, and the exact mechanism is not well understood. As with all malignancies, the pathway for carcinogenesis is likely to be complex and multifactorial. Further studies would be required in order to elucidate the precise histogenesis for multiple secondary neoplasms arising within teratomatous and horseshoe kidneys.

## Conclusion

Synchronous primary carcinoid tumor and primary adenocarcinoma arising within mature teratoma of horseshoe kidney is a unique occurrence. This is the first reported case to our knowledge of mature cystic teratoma coexisting with carcinoid tumor and adenocarinoma in the same kidney. This unique case report emphasizes the need for thorough sectioning and entire submission for histologic evaluation of mature cystic teratomas since multiple additional histogenetically distinct small neoplasms occurring in a mature cystic teratoma could easily be missed through incomplete sectioning and histologic evaluation, potentially compromising patient care. Although longterm follow-up was lacking in the case herein presented, this unique occurrence of three neoplasms in horseshoe kidney was not associated with local recurrence and metastasis, was surgically curable, and had a rather favorable prognosis.

## Consent

Consent was received from the patient before publication.

## Competing interests

The authors declare that they have no competing interests.

## Authors' contributions

HBA participated in the histopathological evaluation, performed the literature review, acquired photomicrographs, and drafted the manuscript. AMP and AVP conceived and designed the study, gave and reviewed the final histopathological diagnosis, and revised the manuscript for important intellectual content. All authors read and approved the final manuscript.
